# Long-term stability of PVDF-SiO_2_-HDTMS composite hollow fiber membrane for carbon dioxide absorption in gas–liquid contacting process

**DOI:** 10.1038/s41598-023-31428-8

**Published:** 2023-04-04

**Authors:** Honglei Pang, Yayu Qiu, Weipeng Sheng

**Affiliations:** 1 Nanjing Vocational University of Industry Technology, Nanjing, 210023 People’s Republic of China; 2 Nanjing Vocational College of Information Technology, Nanjing, 210023 People’s Republic of China; 3 Zhejiang Xinchai CO., LTD, Shaoxing, 312500 People’s Republic of China

**Keywords:** Environmental sciences, Environmental chemistry, Pollution remediation

## Abstract

Hybrid polyvinylidene fluoride-silica-hexadecyltrimethoxysilane (PVDF-SiO_2_-HDTMS) membranes were fabricated via a non-solvent-induced phase-inversion method to create stable hollow-fiber membranes for use in the membrane contact absorption of carbon dioxide (CO_2_). The surface properties, performance characteristics, and long-term performance stability of the prepared membranes were compared and analyzed. The outer surfaces of the prepared membranes were superhydrophobic because of the formation of rough nanoscale microstructures on the surfaces and their low surface free energy. The addition of inorganic nanoparticles improved the mechanical strength of the PVDF-SiO_2_-HDTMS. Long-term stable operation experiments were carried out with a mixed inlet gas (CO_2_/N_2_ = 19/81, v/v) at a flow rate of 20 mL/min. The absorbent liquid in these experiments was 1 mol/L diethanolamine (DEA) at a flow rate of 50 mL/min. The mass transfer flux of CO_2_ through the PVDF-SiO_2_-HDTMS membrane decreased from an initial value of 2.39 × 10^–3^ mol/m^2^s to 2.31 × 10^–3^ mol/m^2^s, a decrease of 3% after 20 days. The addition of highly stable and hydrophobic inorganic nanoparticles prevented pore wetting and structural damage to the membrane. The PVDF-SiO_2_-HDTMS membrane was found to have excellent long-term stable performance in absorbing CO_2_.

## Introduction

Biogas is a promising renewable energy source^1^ that is mainly composed of CH_4_ (55–65%), CO_2_ (30–45%)^2^, and other trace gases. In order to use it as a fuel, its content of CH_4_ must be at least 95%, so the absorption of carbon dioxide (CO_2_) is of great significance for the practical application of biogas. Carbon dioxide (CO_2_) capture from biogas by hollow-fiber membrane contactor systems have been investigated by several researchers. In a membrane contactor system for CO_2_ absorption, the mixed gas and the liquid absorbent flow on opposite sides of the hollow-fiber membrane, and the CO_2_ in the gas is absorbed by the liquid absorbent after passing through the hollow-fiber membrane. The hollow-fiber membrane acts as a non-selective barrier between the liquid phase and the gas phase, separating the gas phase from the liquid phase and providing a large gas–liquid contact area. Studies have shown that in the process of membrane contact absorption, the absorbing liquid enters and wets the membrane pores^3–5^. The diffusion rate of CO_2_ in the gas is greater than in the liquid phase. This greatly increases the diffusion resistance of the membrane to CO_2_, resulting in a rapid decrease in the mass transfer flux of CO_2_. The hollow-fiber membrane must therefore be made hydrophobic.

During a membrane contact absorption process, the membrane will inevitably be wetted because the pressure of the liquid phase must be higher than the pressure of the gas phase to avoid the generation of bubbles. The transmembrane pressure difference thus drives absorbent into the pores, which wets the pores. By contrast, when a chemical absorbent is used, the surface properties (pore size, porosity, roughness, and chemical composition) of the membrane change because of the susceptibility of the polymer membrane material to erosion by alkaline liquids^6–8^. This increases the pore size of the membrane and decreases the surface contact angle, thus decreasing the hydrophobicity of the membrane. In addition, during long-term operation, vaporized liquid absorbent enters the membrane pores and wets them as the vapor condenses. Recent research has therefore focused on the development of superhydrophobic hollow-fiber membranes for the long-term stable operation of membrane contact absorption processes^9,10^.

In our previous studies, superhydrophobic hybrid polyvinylidene fluoride-hexadecyltrimethoxysilane (PVDF-HDTMS) membranes were fabricated via a non-solvent-induced phase-inversion method with a mixture of ammonia and water as the non-solvent additive and dehydrofluorination reagent and HDTMS as the hydrophobic modifier. A long-term CO_2_ membrane contact absorption experiment was conducted to investigate the long-term stability of the membrane under moderate alkaline conditions. The CO_2_ mass transfer flux of the PVDF-HDTMS membrane contactor was found to have decreased by only 17% and then remained stable over 17 days of membrane contact absorption with 1 mol/L diethanolamine (DEA) as the absorbent. Although the PVDF-HDTMS membranes were found to have the excellent long-term stability, current research suggests that further improvements are possible.

Studies have shown that using special methods to deposit hydrophobic inorganic nanoparticles on hollow-fiber membranes can result in superhydrophobic hollow-fiber membranes with excellent long-term stability^11,12^. Zhang et al.^13^ developed a highly hydrophobic organic–inorganic composite hollow-fiber membrane by incorporating a fluorinated silica inorganic (fSiO_2_) layer on a polyetherimide organic membrane substrate via a sol–gel process. The membrane contactor remained fairly stable over 31 days’ operation using a 2M aqueous sodium taurinate solution as the absorbent, with a 20% decrease from the initial CO_2_ flux. This is mainly because the incorporation of the fSiO_2_ inorganic layer resulted in high hydrophobicity and protected the polymeric substrate from attack by the chemical absorbent, thus increasing the lifetime of the membrane. Xu et al.^14^ fabricated an inorganic–organic fluorinated titania-silica (fTiO_2_–SiO_2_)/polyvinylidene fluoride (PVDF) composite membrane by forming a superhydrophobic SiO_2_–TiO_2_ inorganic layer on the PVDF membrane substrate via facile in situ vapor-induced hydrolyzation followed by hydrophobic modification. The CO_2_ absorption flux of the fTiO_2_–SiO_2_/PVDF composite hollow-fiber membrane decreased by merely 10% over the 31 days of the long-term test because its high chemical resistance and hydrophobicity effectively prevented corrosion of the PVDF substrate by the chemical absorbent monoethanolamine (MEA). This shows that the incorporation of hydrophobic inorganic nanoparticles into hollow-fiber membranes can increase the hydrophobicity and corrosion resistance of the membrane, thus improving the long-term performance of the membrane.

In this study, we describe the design and fabrication of polyvinylidene fluoride-silica-hexadecyltrimethoxysilane (PVDF-SiO_2_-HDTMS) composite membranes via a simple non-solvent-induced phase-inversion method using an ammonia-water mixture as a non-solvent additive and dehydrofluorination reagent, HDTMS as a hydrophobic modifier, and nano-SiO_2_ particles as inorganic filler. The physical and chemical properties of the prepared PVDF-SiO_2_-HDTMS film were characterized and the long-term stability for CO_2_ absorption was examined using DEA as an absorbent.

## Experimental

### Formation mechanism of the PVDF-SiO_2_-HDTMS membranes

**Figure 1:**
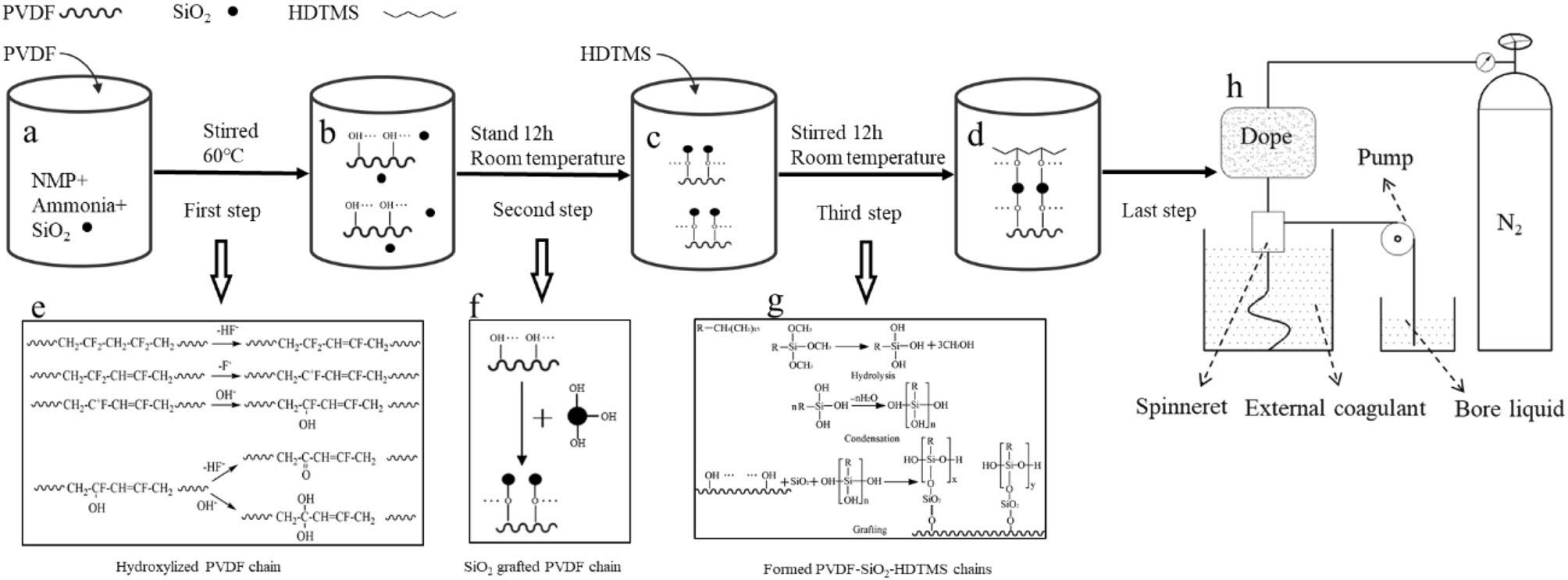
Mechanism of forming the PVDF-SiO_2_-HDTMS chains.

PVDF-SiO_2_-HDTMS membranes were fabricated in four steps. The first step (Fig. 1a,b) was to introduce oxygen-containing functional groups into PVDF molecules through defluorination and oxygenation reactions^15^ (Fig. 1e). In the second step (Fig. 1b,c), the hydroxyl groups on the PVDF chains reacted to form –O– bonds with most of the hydroxyl groups on the surface of the SiO_2_, thus forming PVDF-SiO_2_ dope (Fig. 1f). In the third step (Fig. 1c,d), HDTMS was added to the polymer solution, where it was hydrolyzed by the small amount of water in the solution to form silanol. Polycondensation then occurred between silanol molecules to form polysiloxane, which subsequently reacted with the hydroxyl groups in PVDF-SiO_2_.The mechanism by which HDTMS is grafted onto PVDF-SiO_2_ is shown in (Fig. 1g), where R represents a hexadecyl group. In the last step (Fig. 1h), PVDF-SiO_2_-HDTMS hollow-fiber membranes were fabricated by a non-solvent-induced phase-inversion process.

### Materials

PVDF L-6020 in the form of pellet particle was purchased from Solvay Advance Polymers, USA, and used for the fabrication of the hollow fiber membranes. The reagents of N-methyl-2-pyrrolidone (NMP, ≥ 99.0% purity), ammonia water (25–28% purity, pH = 12–13), diethanolamine (DEA, 99.0% purity), and ethanol (≥ 99.7% purity) were supplied by Chengdu Kelong Inc., China. HDTMS was supplied by Aladdin Inc., China. Nano-SiO_2_ particles (The average particle size of is 50 nm, Hydrophilic) were supplied by Shanghai Macklin Biochemical Co., Ltd., China.

### Fabrication of hollow fiber membrane and membrane contactor module

PVDF-SiO_2_-HDTMS hollow-fiber membranes were fabricated via a dry-jet wet-spinning phase-inversion method. The PVDF and SiO_2_ were dried in a vacuum oven at 70 ± 2 °C for 24 h, after which the dehydrated PVDF polymer particles were gradually added to a magnetically stirred mixture of NMP, ammonia, water, and SiO_2_ at 60 °C until the particles were completely dissolved. During this process, the solution gradually turned brown. After the solution was allowed to stand for 12 h, HDTMS was added and stirred at room temperature for another 12 h to form a homogenous dope. The dope was degassed under vacuum overnight before spinning. The compositions of each dope used in this study are shown in Table 1. The parameters of the spinning process are shown in Table 2. The fabricated hollow-fiber membranes were immersed in pure ethanol for 15 min immediately after the spinning process, subsequently stored in water for 3 days to remove NMP and additives, and then immersed in methanol for one day to protect the formed pores. Finally, the membranes were maintained at room temperature to evaporate the residual methanol. The performance of the membrane was compared to that of a previously described PVDF-PA-8 membrane that was fabricated without amine water treatment and the addition of inorganic nanoparticles^16^.

**Table 1:** Polymer dope composition.

Membrane	NMP (g)	PVDF (g)	Ammonia (g)	HDTMS (g)	SiO_2_ (g)	PA (g)	External coagulant	Bore liquid
PVDF-SiO_2_-HDTMS	80	20	1	1.5	1.5	0	Ethanol	Distilled water
PVDF-HDTMS^17^	80	20	1	1.5	0	0	Ethanol	Distilled water
PVDF-PA-8^16^	74	18	0	0	0	8	Distilled water	NMP/H_2_O = 80/20

**Table 2:** Hollow fiber spinning parameters.

Spinning parameter	Value
Dope extrusion rate (ml/min)	4.5
Bore liquid (wt. %)	Distilled water
Bore liquid flow rate (ml/min)	1.7
Air gap distance (cm)	0
Spinneret (od/id) (mm)	1.6/0.8
Spinneret wall thickness (mm)	0.3
Spinning dope temperature (°C)	25
Coagulant temperature (°C)	25
Bore liquid temperature (°C)	25

### Characterization

The surface functional groups of the membranes were determined by the attenuated total reflectance-Fourier transform infrared spectroscopy (ATR-FTIR) on a NEXUS 870, NICOLET, USA. The ATR-FTIR spectra were collected over a scanning range of 500–4000 cm^−1^ with 16 scans over 4.0 cm^−1^.

The morphology images and the EDS spectrum and mapping of the hollow fibers were observed by the scanning electron microscopy with an energy-dispersive X-ray spectrometer (EDS) (Tabletop Microscope FEI Quanta 250 FEG, USA). The cross section was obtained by fracturing each membrane in liquid nitrogen. The samples were sputtered with gold for 15 s at 40 mA current before the test. The samples of the EDS were sputtered with gold for 15 s at 40 mA current before the test.

A sessile drop technique using a goniometer (Shanghai Zhongchen Digital Technology Instrument Corporation Limited, JC2000D1, China) was used to measure the contact angles of the outer surface of the membranes. For each measurement, 3 μL of pure water or 1M DEA was pumped out from a syringe. The liquid drop remained on the membrane outer surface for 3 min before recording, and the result was adopted as the average value of ten measurements for each sample.

The nitrogen (N_2_) gas permeation test was conducted to obtain the mean pore size and effective surface porosity. The wettability resistance of the prepared membrane was assessed by measurements of the critical water entry pressure (CEPw). The measurement method of the nitrogen (N_2_) gas permeation and CEPw were referred to literature^17–19^.

The tensile strength and elongation at break were measured using a tensile test device (Japan Shimadzu AGS-J) to evaluate the mechanical properties of the membranes. The test was conducted at room temperature and the testing speed was set at 10 mm/min^16^.

### CO_2_ contact absorption experiment

CO_2_ absorption experiments process and the experimental setup could refer to our previous study^17^. The corresponding parameters for the experiments are shown in Table 3. Before each test, the system was operated for at least 30 min to obtain a steady state. The CO_2_/N_2_ mixture (19 vol.% CO_2_) was served as the feed gas flowing through the lumen side of the membranes, and the aqueous DEA solution of 1 mol/L was employed as the liquid absorbent flowing through the shell side of the membranes counter-currently. The flowrate of inlet gas was controlled at 20 mL/min by a gas rotameter, and the flowrate of absorption liquid was controlled by a constant flow pump at 50 mL/min. In order to prevent gas bubbles into the liquid phase, the pressure in the liquid side was controlled 20 kPa higher than that in the gas side. The concentrations of CO_2_ in the inlet and outlet gas were measured by the CO_2_ detector (MIC-800, Shenzhen Yiyuntian electronics technology Co. Ltd., China). The equation to calculated CO_2_ absorption flux of the PVDF membranes referred to our previous article^17^.

**Table 3:** Characteristics of membrane contactors.

Parameter	Value
Module length (mm)	200
Module inner diameter (mm)	8
Effective fiber length (mm)	150
Number of fibers	4
Fiber inner diameter (mm)	0.6–0.7
Fiber outer diameter (mm)	1.1–1.2
Gas–liquid flow	Counter-current flow, gas through the lumen, liquid through the shell

## Results and discussion

### Membrane surface chemical structure

**Figure 2:**
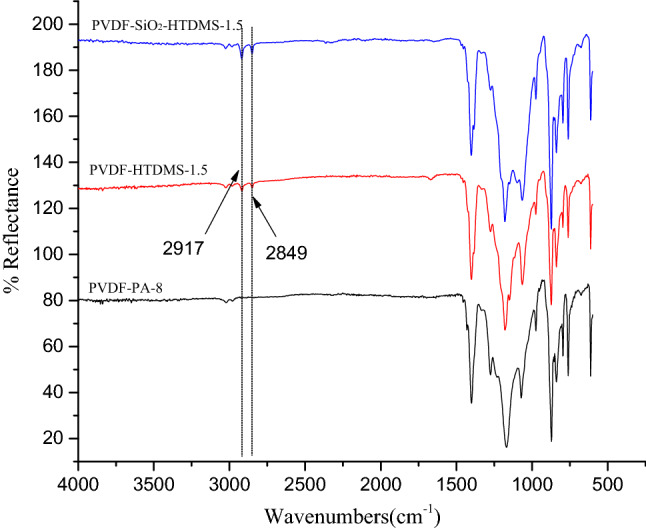
ATR-FTIR spectra of the membranes.

Figure 2 shows the ATR-FTIR spectra of the outer layers of the fabricated membranes and of the PVDF-PA-8 membrane (without the grafting treatment described in our previous work^16^). Compared with the PVDF-PA-8 membrane, two new bands were observed at 2917 and 2849 cm^−1^ in the ATR-FTIR spectra of the PVDF-SiO_2_-HDTMS and PVDF-HDTMS membrane outer layers. These bands are ascribed to the stretching of methylene groups (CH_2_) in the HDTMS chains and show that HDTMS was successfully grafted onto the outer surfaces of the PVDF-SiO_2_-HDTMS and PVDF-HDTMS membranes. The absence of a signal from the Si–O–Si functional groups on the outer surface of the PVDF-SiO_2_-HDTMS membrane may be due to its characteristic band coinciding with that of PVDF.

### Morphology of a hollow fiber membrane

**Figure 3:**
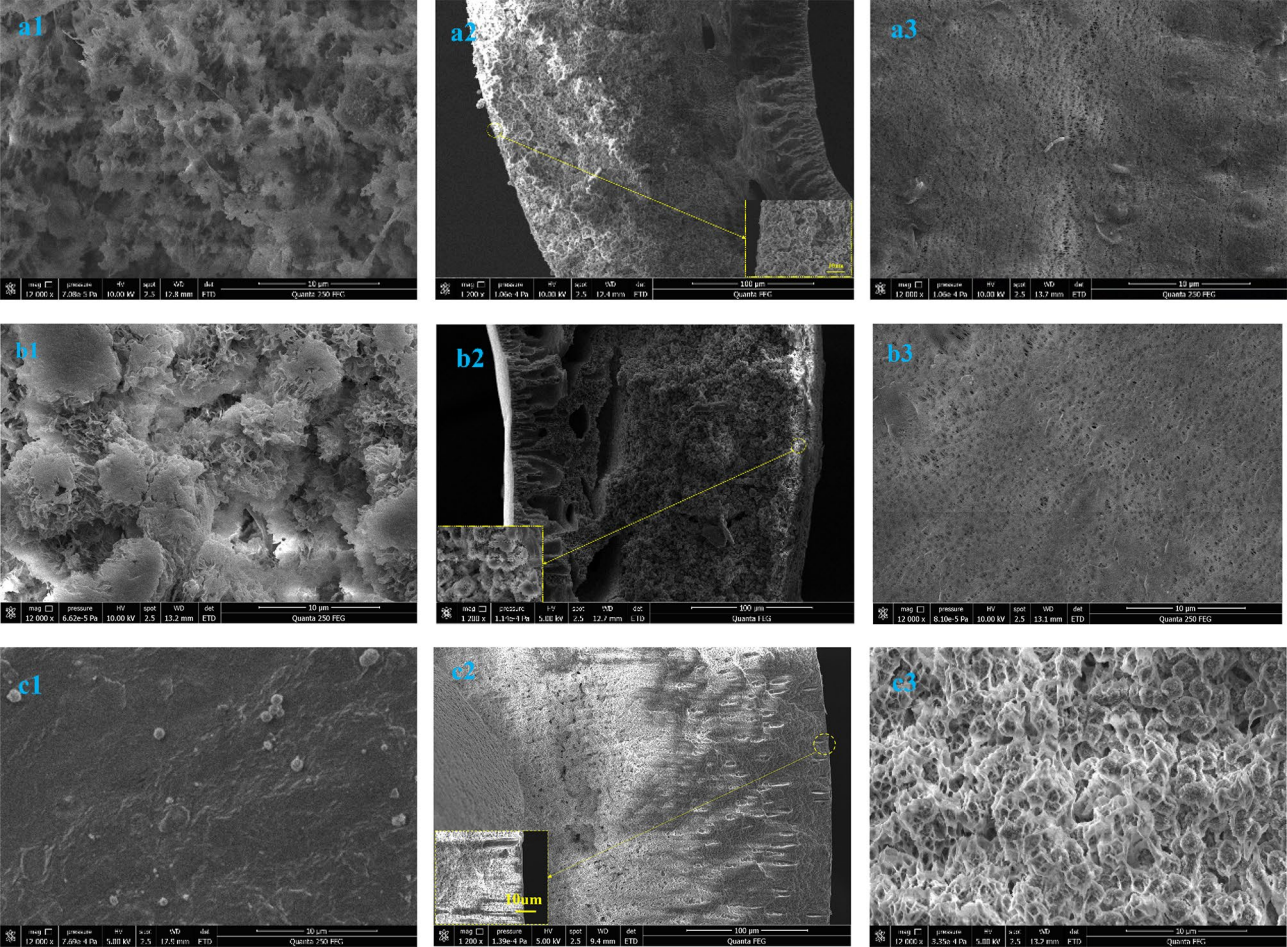
SEM images of the hollow fiber membranes. (**a**) PVDF-SiO_2_-HDTMS; (**b**) PVDF-HDTMS; (**c**) PVDF-PA-8; (1) outer surface; (2) cross section; (3) inner surface.

Figure 3 shows SEM images of the prepared hollow-fiber membranes and PVDF-PA-8 membrane. For PVDF, a semi-crystalline polymer, the wet process for the preparation of the hollow-fiber membranes results in the membrane being formed mainly by liquid–liquid or solid–liquid stratification. The bath conditions play an important role in determining the membrane structure^20,21^. Pure ethanol was used as the external coagulation bath and water as the core fluid in the preparation of the PVDF-SiO_2_-HDTMS and PVDF-HDTMS membranes. In the PVDF-PA-8 membrane preparation process, water and 80% NMP aqueous solution were respectively used as the external coagulation bath and the core fluid. Figure 3 shows that the PVDF-SiO_2_-HDTMS and PVDF-HDTMS membranes had no outer skin layers but that they each had an inner skin layer. The outer surfaces of the PVDF-SiO_2_-HDTMS and PVDF-HDTMS membranes were mainly composed of spherulitic structures, while the inner and outer skin structures of PVDF-PA-8 are just the opposite. This was mainly due to the use of “soft” pure ethanol and 80% NMP aqueous solution in the coagulation bath. The exchange rate between solvent and non-solvent is lower during the phase transfer during the spinning process than after the polymer crystallization process, so solid–liquid stratification leads to form spherulite structures.

Further observation revealed that the outer surface of the PVDF-HDTMS membrane had a honeycomb spherulitic structure (Fig. 3b), while the spherulite structure of the PVDF-SiO_2_-HDTMS membrane outer surface was covered with lumps (Fig. 3a) that may have been caused by the accumulation of a large amount of SiO_2_.

**Figure 4:**
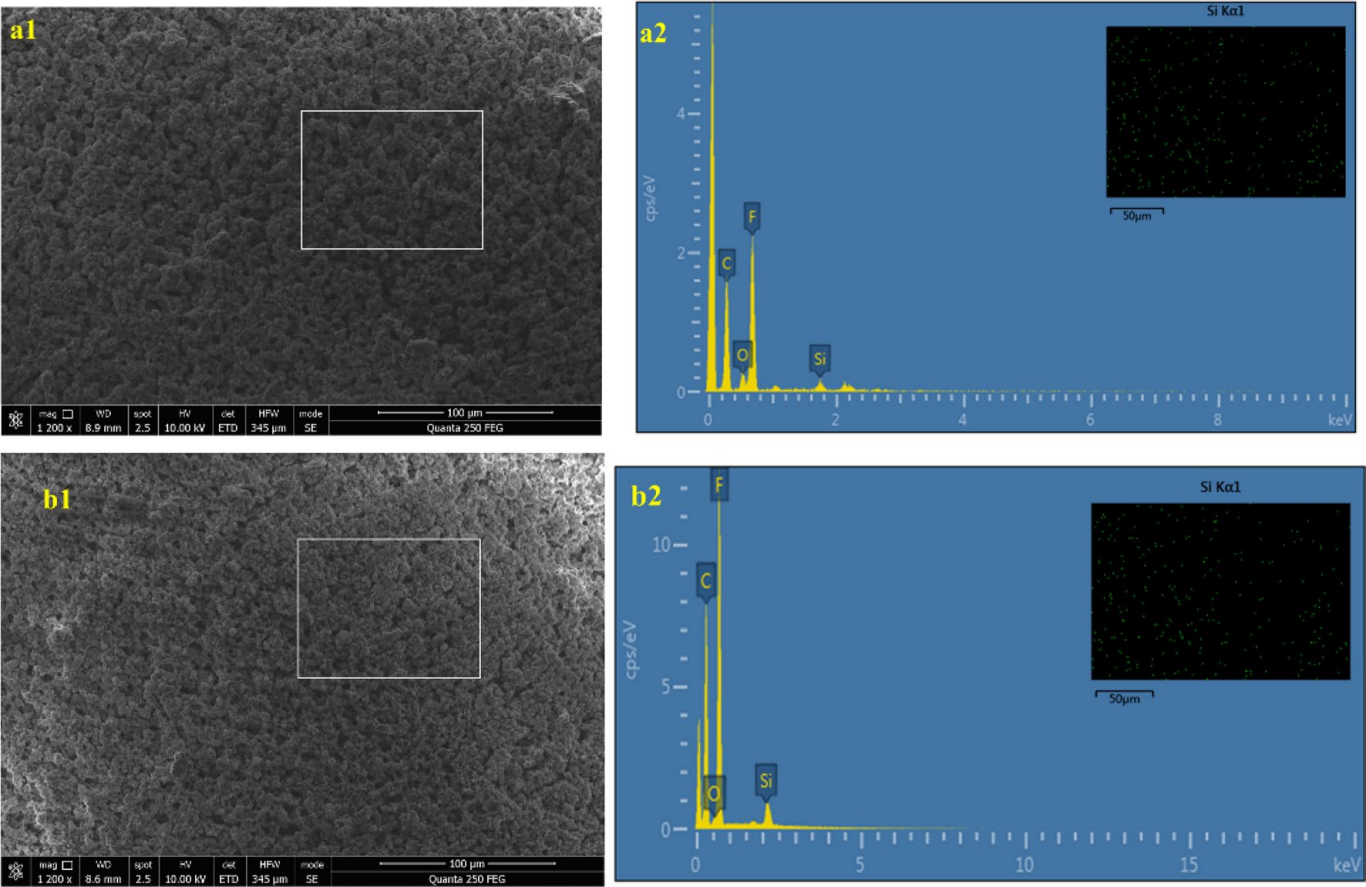
SEM images and the EDS spectrum and mapping (Si) of the membrane. (**a**) PVDF-SiO_2_-HDTMS membrane; (**b**) PVDF-HDTMS membrane; (**a1**), (**b1**) the outer surface image; (**a2**), (**b2**) EDS spectrum of the outer surface and EDS mapping for Si on outer surface.

Because PVDF is hydrophobic and nano-SiO_2_ is hydrophilic, SiO_2_ will disperse poorly and agglomerate in the casting solution upon blending as a result of the interfacial tension between the hydrophobic and hydrophilic inorganic materials^22^. The EDS analysis of the PVDF-SiO_2_-HDTMS and PVDF-HDTMS membrane was displayed in Fig. 4, according to the EDS spectra, it can be found that Si element was the evenly distributed on the outer surface (the results of EDS analysis are consistent with those of ATR-FTIR). Therefore, the SiO_2_ distribution on the cross-section of the PVDF-SiO_2_-HDTMS membrane is uniform, and Fig. 3 shows that no significant agglomeration was observed. This was due to the introduction of hydroxyl groups into the PVDF chains in the ammonia–water mixture^22^, which resulted in enhanced affinity between the PVDF chains and the hydrophilic nano-SiO_2_ particles.

### Gas permeability and hydrophobicity of the hollow fiber membranes

The gas permeabilities of the membranes were determined using N_2_ permeation tests (Table 4), as were previous results for the PVDF-PA-8 membrane^16^. And the data of N_2_ permeation can be viewed in supplementary material 1. The PVDF-PA-8 membrane had the lowest N_2_ permeability of the samples tested. This is because the large number of sponge-like structures in its cross-section increase the tortuosity of the PVDF-PA-8 membrane relative to the other two membranes^23^. The decrease in the nitrogen permeability of the PVDF-SiO_2_-HDTMS membrane may be due to the addition of inorganic nanoparticles that blocked the membrane pores, resulting in the N_2_ permeability of the PVDF-SiO_2_-HDTMS membrane being slightly lower than that of the PVDF-HDTMS membrane. For a porous asymmetric membrane, the overall gas permeance through the membrane can be treated as a combination of Poiseuille flow and Knudsen flow^24^. Assuming that the pores in the skin layer of the asymmetric membranes are cylindrical and plotting N_2_ permeance versus mean pressure, the mean pore sizes and the effective surface porosities over the pore lengths of the membranes are respectively given by the intercept and slope of the plot^25^. The calculated effective surface porosities and average pore sizes of the membranes are shown in Table 4. The surface pore sizes and surface porosities of the PVDF-SiO_2_-HDTMS and PVDF-HDTMS membranes were higher than those of PVDF-PA-8 because the open membrane structure without an outer skin provides larger pore size and surface porosity. The parameters CEP_W_ and outer surface contact angle, which are correlated to the long-term stability of the membranes, are also shown in Table 4. Compared with the PVDF-HDTMS membrane, the PVDF-SiO_2_-HDTMS membrane had significantly higher CEP_W_ and a higher outer surface contact angle. Therefore, the PVDF-SiO_2_-HDTMS membrane is preferable for CO_2_ absorption in membrane contactors.

**Table 4:** Characteristics of the hollow fiber membranes.

Membrane	Mean pore size (nm)	Effective surface porosity (m^−1^)	Elongation at break (%)	Tensile strength (MPa)	CEP_W_ (× 10^5^) (Pa)	Contact angle (°) (water)	Contact angle (°) (DEA)
PVDF-SiO_2_-HDTMS-1.5	20.21	338.8	25 ± 0.4	3.2 ± 0.3	9 ± 0.5	160 ± 0.2	158 ± 0.2
PVDF-HDTMS-1.5	38.93	404.8	15 ± 0.3	1.7 ± 0.3	7.5 ± 0.5	150.0 ± 0.3	146.5 ± 0.5
PVDF-PA-8	17.40	113.1	46.4 ± 0.9	4.1 ± 0.3	9.0 ± 0.5	78.6 ± 0.4	71.5 ± 0.5

During the preparation process, the strength of the PVDF chain decreased because of defluorination by ammonia, resulting in the fabricated membranes having lower mechanical strength. Therefore, as shown in Table 4, the tensile strengths and elongations at break of the PVDF-SiO_2_-HDTMS and PVDF-HDTMS membranes were lower than those of the PVDF-PA-8 membrane. However, the tensile strength and elongation at break of the PVDF-SiO_2_-HDTMS membrane were higher than those of the PVDF-HDTMS membrane. This is mainly due to the formation of Si–O–Si bonds between SiO_2_ and PVDF and between SiO_2_ and HDTMS, which increased the molecular bonding forces between the three and thereby increased the tensile strength and elongation at break of the PVDF-SiO_2_-HDTMS membrane.

### Long-terms performance of the membranes

**Figure 5:**
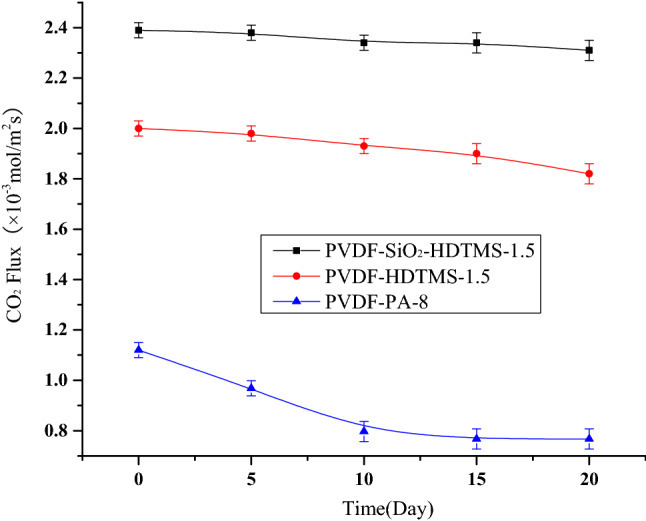
Long-term CO_2_ absorption performance of the membranes (The data of Long-term CO2 absorption performance of the membranes can be viewed in Supplementary material 2).

A long-term CO_2_ membrane contact absorption experiment was conducted using 1 mol/L DEA as the absorbent to investigate the long-term stability of the membranes under moderately alkaline conditions. The results are shown in Fig. 5. The PVDF-PA-8 membrane was completely wetted after 10 days, and the mass transfer flux was reduced by 31.5%. The CO_2_ mass transfer flux of the PVDF-HDTMS membrane decreased by 17% after 20 days. The PVDF-SiO_2_-HDTMS membrane showed excellent long-term stability even in alkaline solution: the CO_2_ mass transfer flux decreased from an initial value of 2.39 × 10^−3^ mol/m^2^s to 2.31 × 10^−3^ mol/m^2^s, a reduction of only 3%.

The ability of the PVDF-PA-8 membrane to withstand wetting is mainly determined by its large cross-sectional tortuosity and its small surface pore size. However, the membrane’s wetting resistance to alkaline solution is poor because of its low hydrophobicity, which results in poor long-term stability.

The PVDF-SiO_2_-HDTMS and PVDF-HDTMS membranes are similar in terms of parameters such as average pore size, surface effective porosity, and surface contact angle, so their anti-wetting performance and air permeability are similar. Little difference was initially observed in these membranes’ mass fluxes of CO_2_. However, over the long term, the performance of the PVDF-HDTMS membrane degraded more than that of the PVDF-SiO_2_-HDTMS membrane. This may be related to the tensile strengths of the membranes. Although the tensile strength of PVDF-HDTMS membrane was sufficient for use in membrane contact absorption, it was lower than that of the PVDF-SiO_2_-HDTMS membrane. In the course of long-term operation, the PVDF-HDTMS membrane eroded more in the alkaline solution, leading to more rapid performance degradation.

### The chemical stability of the composite membrane

In the process of membrane contact absorption, long-term contact between the membrane and the chemical absorbent results in a chemical reaction occurring between the surface of the membrane and the chemical absorbent, causing deterioration in the long-term stability of the membrane and decreasing its CO_2_ mass transfer flux. To verify the long-term corrosion resistance of the PVDF-SiO_2_-HDTMS hollow-fiber membranes to chemical absorbents, nitrogen permeation (The data of N_2_ permeation can be viewed in supplementary material 3) and contact angle experiments were conducted to characterize the performance of the hollow-fiber membranes after 20 days’ stable operation.

The surface pore size and porosity of the three types of membranes after 20 days’ operation are shown in Table 5. The surface pore size and porosity calculations (Table 5) showed large changes in the surface pore sizes and porosities of the three types of membranes. This indicates that DEA did not have much effect on the surface morphologies of the membranes. The contact angles of the three types of membranes decreased after 20 days’ long-term operation in the order PVDF-SiO_2_-HDTMS < PVDF-HDTMS < PVDF-PA-8. The work of Sadoogh et al.^26^ suggests that the degradation of PVDF membrane performance may be due to chemical degradation of the interface between PVDF and MEA, which leads to the dehydrogenation and fluorination of the PVDF surface. Therefore, the reason for the smaller decrease in the contact angle of PVDF-SiO_2_-HDTMS was that the large amounts of chemically stable, superhydrophobic SiO_2_ grafted onto the surface of the PVDF-SiO_2_-HDTMS membrane effectively prevented the PVDF substrate from contact with the DEA solution. The corrosion in the medium ensured the long-term stable operation of the membrane. Xu et al.^14^ prepared membranes with superhydrophobic f-TiO_2_–SiO_2_ layers on their outer surfaces, while Zhang et al.^13^ prepared membranes with superhydrophobic f-SiO_2_ layers on their outer surfaces. Both groups obtained similar results, which shows that grafting superhydrophobic metal nanoparticles onto the outer surface of the membrane is effective in preventing the chemical corrosion of PVDF by chemical absorbents, thus increasing the chemical corrosion resistance of the membrane.

**Table 5:** Characteristics of the hollow fiber membranes.

Membrane	Mean pore size (nm)	Effective surface porosity (m^−1^)	Contact angle (°) (water)	Contact angle (°) (DEA)
PVDF-SiO_2_-HDTMS	21.02	324.6	154 ± 0.2	148 ± 0.2
PVDF-HDTMS	40.63	385.8	142.0 ± 0.3	126.2 ± 0.3
PVDF-PA-8	20.66	96.5	58.5 ± 0.5	43.5 ± 0.5

In Table 6, the parameters of the PVDF-SiO_2_-HDTMS membrane are compared with those of other hydrophobic membranes reported by other research groups. The carbon dioxide absorption fluxes and long-term stability results in Table 6 clearly show that the PVDF-SiO_2_-HDTMS membrane described in this study is competitive in terms of CO_2_ absorption flux as well as long-term stability when absorbent alkalinity and flow rate are moderate and CO_2_ concentration is low. The PVDF-SiO_2_-HDTMS membrane can maintain high performance and stability while showing excellent CO_2_ absorption flux. The main reason is to graft a large number of superhydrophobic SiO_2_ with high hydrophobicity and chemical stability on the membrane surface, effectively prevent the corrosion of PVDF membrane in in the chemical absorber, and ensure the long-term stable operation of the membrane.

**Table 6:** Comparison of CO_2_ absorption performance in gas–liquid membrane contactor.

References	Membrane types	CO_2_ flux (mol/m^2^s)	Inlet gas type	Absorbent type	Gas flow rate	Absorbent flow rate	Contact angle (°) (water)	Long-term stability
^11^	PVDF + LDPE	3.1 × 10^−3^	20% CO_2_	1M MEA	50 ml/min	Not reported	152 ± 3.2	Decline 14% after 1 day
^14^	PVDF + f-TiO_2_-SiO_2_	8.0 × 10^−3^	Pure CO_2_	1M MEA	Not reported	0.25 m/s	124(dynamic)	Decline 10% after 31 days
^23^	PVDF + 5wt.% MMT	9.73 × 10^−4^	Pure CO_2_	Distilled water	0.5 m/s	Not reported	99 ± 1.5	Not reported
^27^	D-TZ-PAN-20	1.9 × 10^−3^	Prue CO_2_	Distilled water	1500 ml/min	240 ml/min	113 ± 2	Not reported
^28^	PVDF + 6%SMM	5.3 × 10^−3^	Pure CO_2_	Distilled water	100 ml/min	300 ml/min	99 ± 1.50	Not reported
^29^	PEI-fSiO_2_-3h	5.0 × 10^−3^	Pure CO_2_	2M sodium taurinate	Not reported	30 ml/min	124.9 ± 1.4	Decline 20% after 31 days
This work	PVDF-SiO_2_-HDTMS	2.23 × 10^−3^	19% CO_2_	1M DEA	20 ml/min	50 ml/min	160 ± 0.2	Decline 3% after 20 days

## Conclusions

In this study, hydrophilic nano-SiO_2_ was added as an inorganic filler to a casting solution, and ammonia was used as a defluorinating agent to hydroxylate PVDF molecules and generate active sites. With the addition of hydrophilic nano-SiO_2_, the hydroxyl groups formed on the PVDF molecule undergo a dehydration reaction to form PVDF-SiO_2_ polymer chain. HDTMS was then added to modify the hydrophobicity. Superhydrophobic PVDF-SiO_2_ with high tensile strength was prepared by spinning by the NIPS method to create an HDTMS organic–inorganic composite membrane. The prepared membrane had a maximum CO_2_ mass transfer flux of 2.39 × 10^−3^ mol/m^2^s. After 20 days of membrane contact absorption with 1 mol/L DEA as the absorbent, the CO_2_ mass transfer flux of the membrane contactor decreased by only 3%. Large quantities of superhydrophobic, chemicaelly stable SiO_2_ grafted onto the surface of the PVDF-SiO_2_-HDTMS film were effective in preventing the corrosion of the PVDF substrate in DEA solution. The film was superhydrophobic, with strong resistance to wetting and chemical stability.

## Supplementary Information

Supplementary Information.

## Data Availability

The datasets used and/or analysed during the current study available from the corresponding author on reasonable request.
