# Supplementary material for: Long-term stability of PVDF-SiO2-HDTMS composite hollow fiber membrane for carbon dioxide absorption in gas–liquid contacting process
Source: Sci Rep. 2023 Apr 4;13:5531. doi: 10.1038/s41598-023-31428-8 (PMC10073106; doi:10.1038/s41598-023-31428-8)
Supplement: Supplementary file 1 — Supplementary Information. [file 41598_2023_31428_MOESM1_ESM.pdf]

Supplementary material1:N_2 permeance of the hollow fiber membranes

	PVDF-HDTMS-1.5	PVDF-SiO_2-HDTMS-1.5	PVDF-PA-8
100	2.23E-6	9.22E-7	2.67E-7
150	2.29E-6	9.31E-7	2.73E-7
200	2.36 E-6	9.71E-7	2.79E-7
250	2.47E-6	9.99E-7	2.82E-7
300	2.56E-6	1.002E-6	2.89E-7
350	2.68E-6	1.016E-6	2.93E-7

According to the calculation formula proposed in Reference [15-17], table4 can be obtained.

Supplementary material2: Fig.5 Long-term CO_2 absorption performance of the membranes

	PVDF-HDTMS-1.5	PVDF-SiO_2-HDTMS-1.5	PVDF-PA-8
0	2	2.39	1.12
5	1.98	2.38	0.968
10	1.93	2.34	0.797
15	1.9	2.34	0.767
20	1.82	2.31	0.767

Supplementary material3: N_2 permeance of the hollow fiber membranes

	PVDF-HDTMS-1.5	PVDF-SiO_2-HDTMS-1.5	PVDF-PA-8
100	2.23E-6	9.22E-7	2.69E-7
150	2.29E-6	9.31E-7	2.79E-7
200	2.37E-6	9.71E-7	2.83E-7
250	2.48E-6	9.99E-7	2.84E-7
300	2.56E-6	1.002E-6	2.96E-7
350	2.7E-6	1.016E-6	3.01E-7

According to the calculation formula proposed in Reference [15-17], table6 can be obtained.
